# Preparation, Characterization, and Antioxidant Properties of Phycocyanin Complexes Based on Sodium Alginate and Lysozyme

**DOI:** 10.3389/fnut.2022.890942

**Published:** 2022-05-24

**Authors:** Bian-Wen Qiao, Xin-Tong Liu, Chen-Xin Wang, Shuang Song, Chun-Qing Ai, Ying-Huan Fu

**Affiliations:** ^1^School of Food Science and Technology, Dalian Polytechnic University, Dalian, China; ^2^National Engineering Research Center of Seafood, Dalian, China; ^3^School of Light Industry and Chemical Engineering, Dalian Polytechnic University, Dalian, China

**Keywords:** phycocyanin, antioxidation, stability, simulated digestion, electrostatic interactions

## Abstract

In this study, phycocyanin-sodium alginate/lysozyme complex (PC-SLC) was prepared for the first time and characterized by UV spectroscopy, Fourier transform infrared spectroscopy (FT-IR), and circular dichroism spectroscopy (CD). The stability of PC-SLC under light, temperature, pH and simulated gastrointestinal fluid was investigated. The scavenging ability of the complexes against DPPH and ABTS radicals was determined. The results showed that the complex formed by the mass ratio of SA-LZM of 0.1 showed the highest PC encapsulation rate (89.9 ± 0.374%). The combination of SA and LZM changed the secondary conformation of PC. The PC-SLC complex shows an irregular spherical structure and the spheres are clustered together. Compared with phycocyanin (PC), its thermal stability was obviously improved, but it was still greatly influenced by light. It could exist stably in simulated gastric fluid (SGF) for 2 h and be slowly digested in simulated intestinal fluid (SIF), which helped to promote the absorption of nutrients in the intestinal tract. Meanwhile, the complex PC-SLC showed high scavenging ability for DPPH and ABTS radicals. It can be concluded that the complexes have good antioxidant activity. This study provides an idea for the construction of PC delivery system and makes it more widely used in food industry and other fields.

## Introduction

*Spirulina* contains a variety of nutrients, such as vitamins, minerals, protein and γ-polyunsaturated fatty acids, such as linolenic acid (1). In addition, *spirulina* species has antiviral, antibacterial, antifungal and antiparasitic activities. *Spirulina* preparation can improve the intestinal environment by promoting the growth of *Lactobacillus, Bifidobacterium* and other intestinal beneficial bacteria (2). Recently, studies on *Spirulina* have shown that *Spirulina* contains many phenolic compounds, which usually have high antioxidant activity (3). Estrada et al. found that phycobiliprotein (PC) extracted from *Spirulina* also has good antioxidant activity (4).

Because PC contains all essential amino acids, it is often used as a nutritional supplement. The potential benefits of PC for human nutrition and health are significant (5). Because of its fluorescent properties, it is also used as a biochemical tracer in immunoassays for detailed knowledge of the various stages involved in the immunoassay process in animals (6). As a water-soluble natural pigment, it is also widely used as food colorant, cosmetic additive and clinical diagnostic (7). In addition, PC can be used to treat oxidative stress-induced diseases because it has significant antioxidant, liver protection and free radical scavenging properties, such as preventing cisplatin induced nephrotoxicity by inhibiting oxidative stress (8). However, the sensitivity of PC to storage and processing conditions leads to variability, precipitation, and discoloration, which limits its application in different fields. Therefore, how to improve the stability of PC during transportation has been a popular research topic in the field of food.

The degradation of PC depends on the aggregation state of the protein, which is influenced by light, solution temperature, pH value and PC concentration (9, 10). Therefore, in the processing of PC as a natural pigment, it is very important to properly control temperature, pH value and light to improve its stability. At present, the stability of PC can be improved by adding stabilizers. Polysaccharides and polyols have been used to stabilize proteins and as stability agents in the food industry and pharmaceutical formulations due to their good food safety and non-cytotoxicity (11). Faieta et al. (12) showed that sucrose has a significant protective effect on PC. It was confirmed by circular dichroism that the loss of color of PC is closely related to the structural instability of the protein. In addition, microencapsulation technology can be used to improve the stability of PC. Das et al. (13) prepared polymer ultrafine particles by electrospray technology, while maintaining PC antioxidant activity and improving its heat resistance and stability.

Natural polysaccharide sodium alginate (SA) is widely used in the delivery systems of bioactive substances due to its good biocompatibility, low price and gel properties. Since the dissociation constants of mannuronic acid (M) and guluronic acid (G) monomers are 3.38 and 3.65, respectively, SA tends to be negatively charged in a wide pH range (14). Lysozyme (LZM) can be used as a model protein to study the interaction between protein and polysaccharide. Its isoelectric point is about 10.8, so they can form complexes through electrostatic attraction in a wide pH range. Therefore, in this study, we prepared phycocyanin sodium alginate/lysozyme (PC-SLC) complex, in which the main force is the electrostatic force between positively charged lysozyme and negatively charged sodium alginate. The purpose is to improve the processing and storage stability of PC by forming a complex, so the light, temperature, pH and gastrointestinal digestive fluid stability of the prepared PC-SLC were measured. The results showed that the formed complexes could improve the stability of PC. The complex PC-SLC has good antioxidant activity. This study provides an idea for constructing the capsule delivery system of algal cyanobacterial proteins, which is conductive to its application in food industry and other fields.

## Materials and Methods

### Materials

PC was purchased from Binmei Biological Co., Ltd. (Zhejiang, China); LZM (molecular weight 14.7 kDa) was obtained from Shanghai Bioengineering Co. (Shanghai, China); SA was provided by Aladdin Industrial Co. (Shanghai, China); Other chemical reagents used were of analytical grade and commercially available.

### Determination of Relative Molecular Mass of SA

High-performance gel-permeation chromatography (HPGPC) was used to determine the relative molecular mass of SA. The gel chromatography column was TSK-gel G5000PWxl (7.5 mm × 30.0 cm) with a column chamber temperature of 30°C. 0.1 mol/L ammonium acetate buffer solution (pH = 6) was used as the mobile phase with a flow rate of 0.4 ml/min. Using the mobile phase as the solvent of the standard dextran solution, which was injected in a volume of 10 μl. Drawing a linear regression equation with retention time t_R_ as the horizontal coordinate and lgMw as the vertical coordinate.

The prepared solution was filtered through a 0.22 μm filter membrane and the rest of the conditions were the same as those for the determination of the standards. It was measured the retention time of the sample peak and the relative molecular mass of the sample was calculated by substituting the retention time into the standard curve.

### Preparation of Phycocyanin-Sodium Alginate/Lysozyme Complexes

The preparation of PC-SLC was based on a previously published method in the literature with slight modifications (15). Stock solutions of PC (0.24 mg/ml), LZM (10 mg/ml), and SA (1 mg/ml) were prepared with deionized-water. The PC stock solution (1 ml) was added dropwise to LZM solution (1 ml) and mixed well, then 1 ml SA stock solution was added dropwise to PC- LZM mixture with SA: LZM mass ratio of 0.1, and strirred at 25°C for 3 h to prepare the complex. The precipitate was collected by centrifugation at 7,800 g for 30 min and washed several times with deionized water until the washing solution contained no PC. The concentration of PC was tested according to the method in Determination of PC Concentration. Then the precipitate was collected by centrifugation and freeze-dried.

### Determination of PC Concentration

PC concentration was determined according to the literature reported by Bennett and Bogorad (16) with slight modification. The supernatant after centrifugation was collected. Then absorbance values were measured using a microplate reader at 620 and 652 nm (Tecan). The calculation formula of PC concentration is as follows:


$$\begin{array}{l} \left[PC\right]\;=\;\frac{OD_{620}\;-\;0.474\;\times \;OD_{652}}{5.34} \end{array}$$

OD_620_: the optical density at 620 nm; OD_652_: the optical density at 652 nm;

The encapsulation efficiency (EE) was calculated according to the following formula:


$$\begin{array}{l} EE\left(\%\right)\;=\;\frac{W_{1}}{W_{2}}\times \;100 \end{array}$$

W_1_: the mass of PC in embedding system; W_2_: the mass of PC in feed.

### The Characterization of the PC-SLC

#### UV–Visible Spectra

The supernatant after centrifugation was collected. UV-visible spectra were obtained using a Lambda 35 UV-visible spectrometer with a scan range of 200-700 nm (PerkinElmer, Japan).

#### Circular Dichroism Spectra

The precipitate after centrifugation of the complex solution is dissolved with deionized water and diluted. Circular dichroism spectra were collected on a Jasco J-1500 Spectropolarimeter from 320 to 190 nm with a scanning rate of 50 nm min^−1^.

#### FT-IR

Dried samples were grounded in an agate mortar, mixed with KBr in the ratio of 1:50 (w/w), pressed into pellets and analyzed by FT-IR spectrometer (PerkinElmer) in the range of 4,000-400 cm^−1^.

#### Scanning Electron Microscopy

A field-emission scanning electron microscopic image was collected using a JSM-7800F (Japan). The precipitate of the complex solution was collected after centrifugation and freeze-dried to obtain a solid powder. Three to five mg of the sample to be tested and dispersed evenly on a conductive adhesive on the sample stage. After vacuum gold plating, the morphology of the complex at magnifications of 5,000, 10,000, 30,000, and 50,000 was observed by SEM. The operating voltage is 3 Kv.

### Stability Study of PC-SLC

#### pH Buffered Solutions

The stability test method of the complex PC-SLC at pH conditions was performed according to the method of Khan et al. (8) with slight modifications. Buffer solutions with different pH values (3, 5, 7.4, and 8) were prepared by mixing 0.2 mol/L aqueous disodium hydrogen phosphate and 0.1 mol/L aqueous citric acid while monitoring with a pH meter. The prepared PC-SLC was dissolved in different pH buffer solutions and then incubated at 25°C. At predetermined time intervals, 1 ml of supernatant was removed and supplemented with the same volume of fresh buffer solution. The absorbance of the supernatant was measured with a microplate reader (Tecan) at 620 and 652 nm. The cumulative release of PC was calculated with the following equation.


$$\begin{array}{l} Cumulative\;PC\;release\;\left(\%\right)\;=\;\frac{W_{t}}{W_{0}}\;\times \;100 \end{array}$$

W_t_: the amount of PC released at time t; W_0_: the amount of PC in the PC-SLC.

#### Thermal Stability

The method was modified with reference to the supplementary method published in literature (17). PC-SLC solid powder prepared was dissolved in disodium hydrogen phosphate-citrate buffer solution at pH 7.4 and incubated for 30 min (protected from light) at four different temperatures (37, 45, 53, 60 and 70°C). The cumulative release of PC was calculated by referring to the formula in pH buffered solutions.

#### Photostability

The photostability of PC-SLC was evaluated using UV lamp and cabinet. PC-SLC was dissolved in disodium hydrogen phosphate-citrate buffer pH 7.4. The solutions were mixed well and incubated under UVB light conditions and shaded conditions, respectively. At predetermined time intervals, 1 ml of supernatant was removed and supplemented with the same volume of fresh buffer solution. The cumulative release of PC was calculated by referring to the formula in pH buffered solutions.

#### *In vitro* Digestion Study

Based on the method described in literature (18), the *in vitro* gastrointestinal model composed of simulated gastric fluid (SGF) and simulated intestinal fluid (SIF) was used to evaluate the *in vitro* digestion of samples with some modifications.

16.4 ml of dilute hydrochloric acid (9.5-10.5% by volume) was added to 800 ml of water, and then 10 g of pepsin was added. After mixing, deionized water was added to make the volume up to 1 L to obtain simulated gastric fluid (SGF).

PC-SLC dissolved in deionized water was mixed with SGF and incubated in a thermostatic water bath at 37°C for 2 h. The sample solution (200 μl) was taken out every 30 min. The sample solution was filtered through a 0.45 μm filter membrane. The cumulative release was determined as before calculated using the formula in pH buffered solutions for the cumulative release of PC.

The pH of 13.6 mg/ml potassium dihydrogen phosphate solution (250 ml) was adjusted with NaOH (0.1 mol/L) to 6.8. It was mixed with 25 mg/ml trypsin solution (100 ml) and 150 ml distilled water was added to obtain the simulated intestinal fluid (SIF). Reference was made to the method of Li et al. (19) for the configuration of the simulated intestinal fluid.

PC-SLC dissolved in deionized water was mixed with SIF and incubated in a thermostatic water bath at 37°C for 6 h. The other steps remained the same as in the SGF.

### Assay of Antioxidant Activity

#### Scavenging Activity of 2, 2'-azino-bis (3-ethylbenzothiazoline-6-sulfonic Acid Ammonium Salt) (ABTS) Radical

The ABTS free radical scavenging assay was modified from the method reported in the literature (20). ABTS (14 mmol/L) was mixed with K_2_S_2_O_8_ (4.9 mmol/L) in equal volumes and kept away from light at room temperature for 12 h to obtain ABTS radical assay solution. The absorbance reached 0.70 ± 0.02 at 734 nm after ABTS radical assay solution dilution with 0.1 mol/L PBS buffer (pH = 7) before measurement. The sample (10 μl) and PBS (20 μl) were mixed with ABTS detection solution (70 μl), incubated for 6 min, and absorbance was measured at 734 nm. Vc was used as positive control. The ABTS free radical scavenging rate is calculated as follows:


$$\begin{array}{l} Scavenging\;activity\;\left(\%\right)\;=\;\frac{A_{0}-A}{A_{0}}\times 100 \end{array}$$

A_0_: the absorbance of the control reaction; A: the absorbance value of the test samples.

#### Scavenging Activity of 1, 1-Diphenyl-2-picrylhydrazyl Radicals

The determination of DPPH radical scavenging activity was referred to Liang's method with some modifications (21). DPPH (1 mg) was dissolved in anhydrous ethanol (24 ml) and mixed thoroughly. DPPH solution (1 ml) was diluted by adding a certain volume (0.5 ml) of anhydrous ethanol solution, mixed thoroughly and then measured the absorbance value A_0_ (between 0.6 and 1.0) at 519 nm. Add 1 ml of DPPH solution to the EP tube, add x μl of sample solution to the pre-test addition, add (500-x) μl of anhydrous ethanol solution, mix thoroughly, and measure the absorbance value at 519 nm after 30 min of standing. Vc was performed as positive control. The DPPH radical scavenging rate is calculated by the following equation:


$$\begin{array}{l} Scavenging\;activity\;\left(\%\right)\;=\;\frac{A_{0}-A}{A_{0}}\times 100 \end{array}$$

A_0_: the absorbance of the control reaction; A: the absorbance value of the test samples.

### Data Analysis

All data are shown as mean ± standard deviation (M ± SD) of three parallel samples (*n* = 3) within significance *P* < 0.5 after passing single factor analysis of variance SPSS 25.0 software. Significant differences between two groups of data were determined by *t*-test, and One-ANOVA was performed between multiple groups of data.

## Results and Discussion

### Molecular Weight of SA

**Figure 1:**
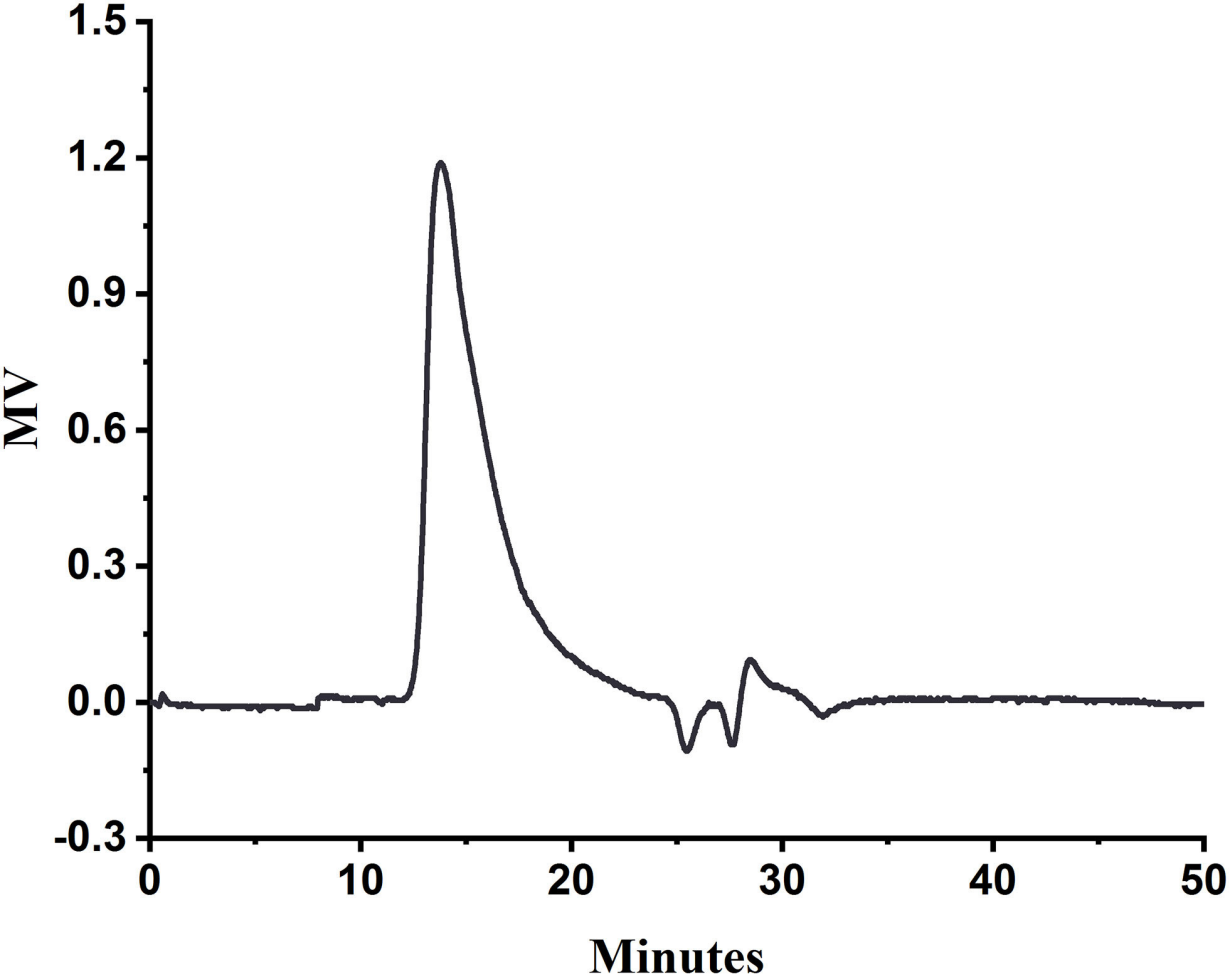
HPGPC of SA.

The HPGPC of SA is shown in Figure 1. The measured retention time of SA in the column was 13.76 min, and the molecular weight of SA was calculated as 4696.8 kDa from the plotted standard curve equation lgMw = 0.2966t_R_ – 10.753 (R^2^ = 0.9986).

### Characterization of PC-SLC

**Figure 2:**
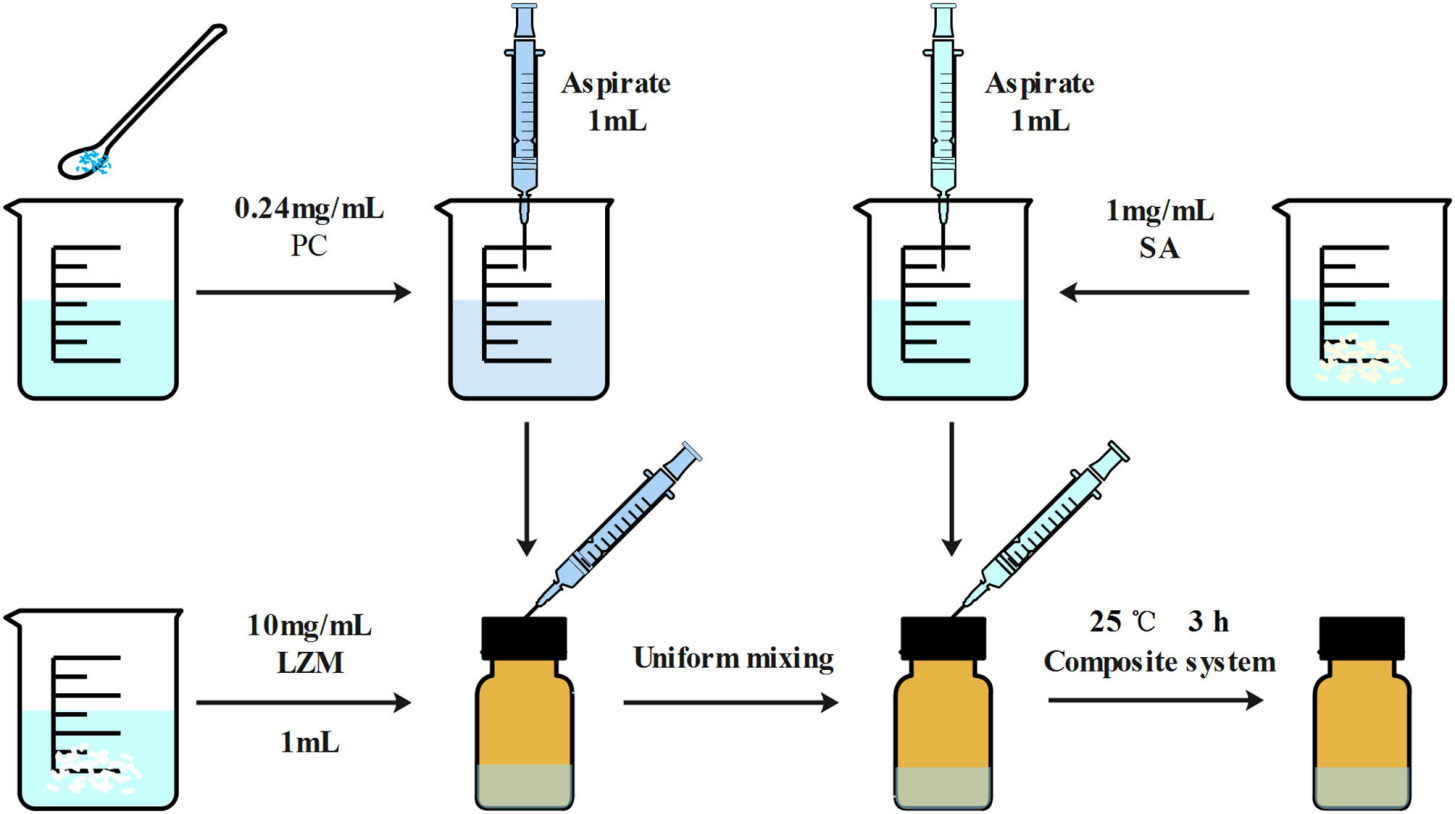
Schematic diagram of the preparation of LZM and SA encapsulated PC particles. The main reason for the formation of particles is due to the electrostatic interactions between the macromolecules in the whole system.

LZM and SA solutions were prepared and mixed at a weight ratio of 10:1 to encapsulate PC to form PC-SLC complex solutions. The specific preparation process is shown in Figure 2.

**Figure 3:**
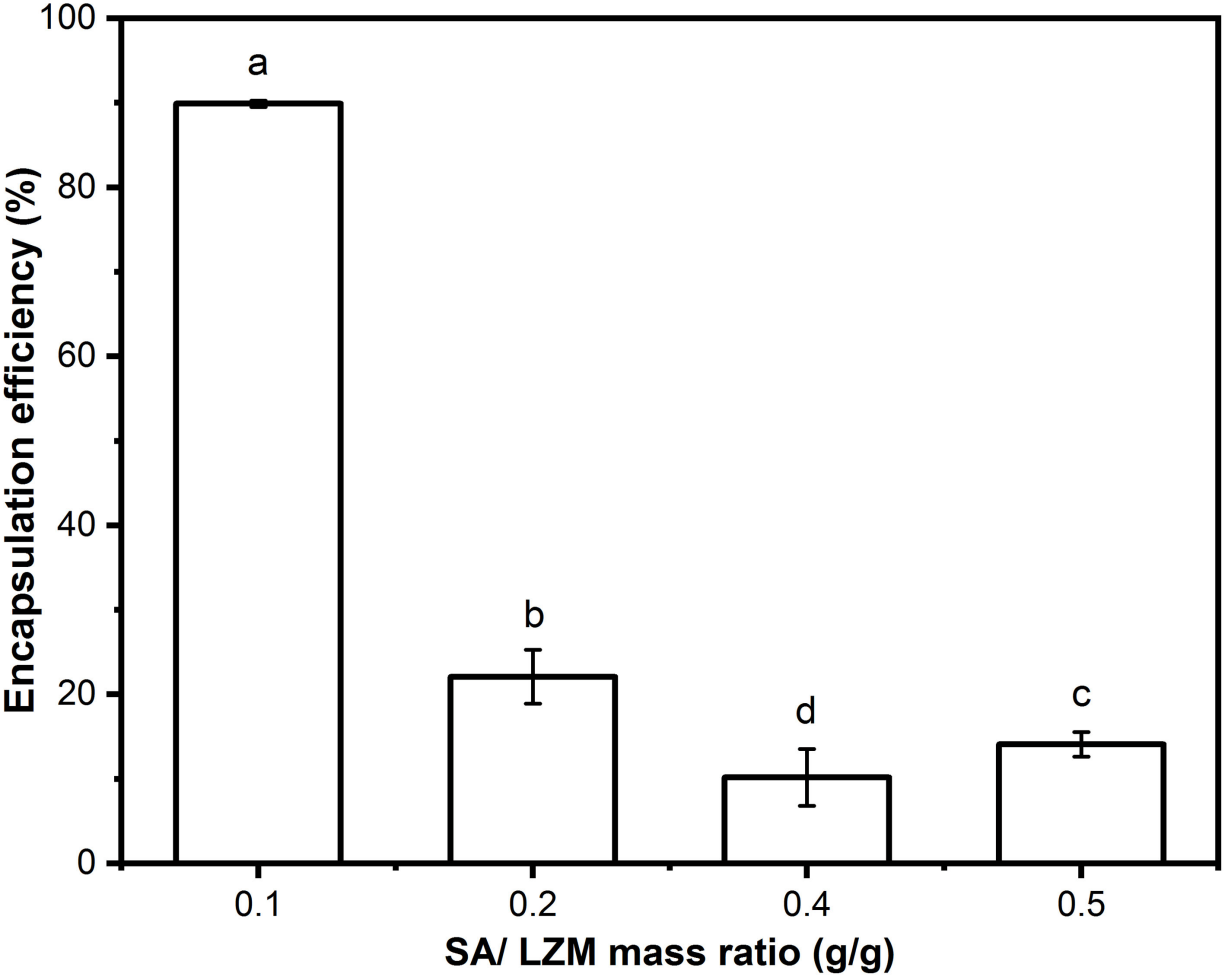
Encapsulation efficiency of PC-SLC formed by different weight ratios of SA/LZM. Different letter a-d values indicate significant differences between the data (*p* < 0.05).

As shown in Figure 3, when the weight ratio of SA to LZM was changed, there was a significant difference in the encapsulation efficiency of PC. The complex PC-SLC formed by the mass ratio of SA-LZM of 0.1 showed the highest encapsulation efficiency of PC (89.9 ± 0.374%). This may be due to the negative charge of PC and SA in aqueous solution, which can produce electrostatic interaction with LZM with opposite charge. It has been shown that electrostatic interactions between polysaccharides and proteins in a system can be achieved by changing the concentration and ratio of both. The results of Wang's study found that mixing different ratios of β-lactoglobulin with pectin in an aqueous system can lead to the formation of cohesions due to electrostatic interactions. It was found that increasing the mixing ratio of β-lactoglobulin could lead to the formation of cohesions with higher elasticity (22). When there was too much SA, there were more anions than cations in the whole system, the formation of PC-SLC may be hindered. Therefore, the follow-up experiments in this study were carried out with the weight ratio of SA to LZM of 1:10.

#### UV–Visible Spectroscopy Analysis

**Figure 4:**
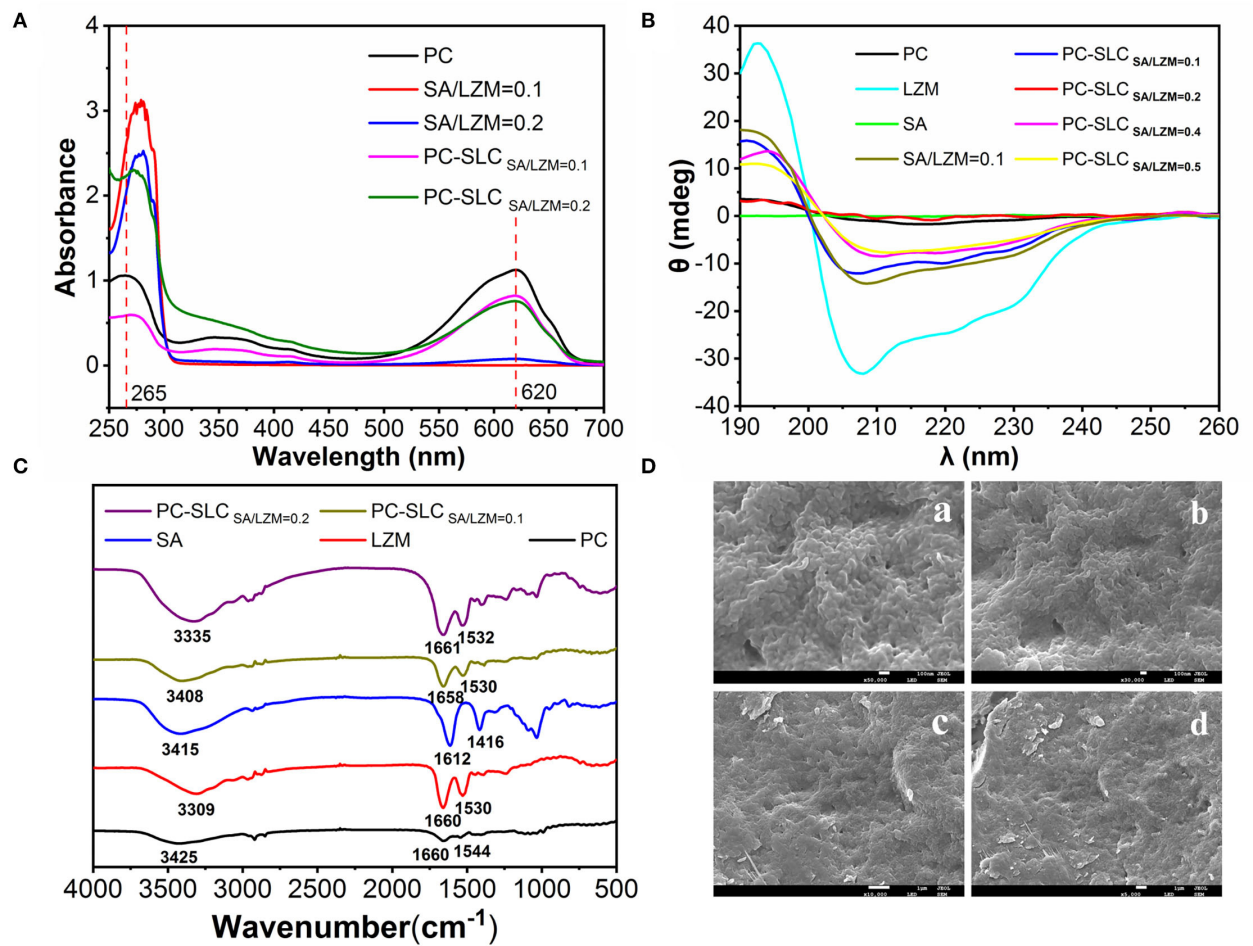
UV spectra **(A)**, circular dichroism **(B)**, FT-IR spectra **(C)** of PC, LZM, SA, different weight ratios of SA/LZM and complex PC-SLC containing different weight ratios of SA/LZM, and different magnifications [50,000x (a), 30,000x (b), 10,000x (c), and 5,000x (d)] of complex PC-SLC Scanning electron micrographs **(D)**.

In this study, the supernatants centrifuged after the formation of PC, SA-LZM, and PC-SLC were measured separately by UV-visible spectroscopy. As shown in Figure 4A, the characteristic absorption peaks of PC appeared at 652 and 620 nm. For the same protein concentration, the lower absorption peak indicates less PC in the supernatant (23), which also indirectly indicates the higher encapsulation efficiency of PC. The weight ratio of SA to LZM is one of the important factors affecting the encapsulation rate of PC. The weakest PC characteristic peak was shown when the weight ratio of SA to LZM in the complex was 0.1. It is thus clear that the complex formed have the highest encapsulation efficiency for PC when the weight ratio of SA to LZM is 0.1.

#### CD Analysis

The CD spectrum of the complex PC-SLC is shown in Figure 4B. The LZM solution has one positive peak at 195 nm and two negative peaks at 208 and 222 nm. After the formation of the complex PC-SLC, the positive peak at 195 nm is slightly shifted and diminished toward the lower wave number and the negative peak at 208 nm is shifted toward the higher wave number. The obtained CD spectra were analyzed using secondary structure estimation software and the results are shown in Table 1. The results showed that the secondary structures of the complexes PC-SA/LZM formed with different weight ratios of SA/LZM were all changed compared to PC (24). Comparing the secondary structures of PC, SA/LZM with a weight ratio of 0.1 and the complex PC-SA/LZM formed from them, it is clear that the combination of SA and LZM resulted in changes in the secondary structure of PC. The combination of SA and LZM resulted in a 25.9% decrease in α-helix deflection, 7.6% increase in β-fold deflection, 1.6% increase in β-turn angle deflection, and 16.7% increase in radon deflection of PC. The β-folding is more flexible, which can improve the flexibility properties and expansion of the protein (25). Comparison with PC shows that the β-folding of the complexes formed by different weight ratios of SA/LZM all changed. And when the weight ratio of SA/LZM was 0.1, the corresponding complexes underwent the greatest increase in deflection of β-folding. The results suggest that the presence of SA and LZM may cause the greatest structural expansion of PC.

**Table 1:** The secondary structure composition of PC, LZM, SA, SA/LZM with a weight ratio of 0.1, and PC-SLC formed by different weight ratios of SA/LZM (0.1, 0.2, 0.4, 0.5).

	**HELIX**	**BETA**	**TURN**	**RANDOM**
PC	50.8 ± 1.8^a^	29.9 ± 1.0^ab^	1.9 ± 2.0^de^	17.4 ± 3.6^c^
LZM	22.5 ± 0.8^cd^	39.9 ± 1.2^a^	9.6 ± 1.6^c^	28 ± 1.3^ab^
SA	18.6 ± 4.5^d^	40.4 ± 11.5^a^	12.5 ± 5.8^bc^	28.5 ± 5.7^ab^
PC-SLC_SA/LZM = 0.1_	24.9 ± 0.4^cd^	37.5 ± 1.9^a^	3.5 ± 0.2^de^	34.1 ± 2^a^
PC-SLC_SA/LZM = 0.2_	35.9 ± 7.5^b^	31.9 ± 5.3^a^	0.9 ± 1.3^e^	31.3 ± 5.2^a^
PC-SLC_SA/LZM = 0.4_	40.9 ± 1.8^b^	17.9 ± 2.1^c^	18.2 ± 1.2^ab^	23 ± 2.1^bc^
PC-SLC_SA/LZM = 0.5_	40.1 ± 0.8^b^	19.4 ± 1.6^bc^	20.1 ± 1.1^a^	20.4 ± 1.2^bc^
SA/LZM = 0.1	27.6 ± 0.8^c^	37.6 ± 0.9^a^	7 ± 1.1^cd^	27.8 ± 2.8^ab^

*Values are expressed as means ± standard deviation; (n = 3); Superscript (a–e) values with different letters indicate significant differences at the same concentration (p < 0.05)*.

#### FT-IR Analysis

FTIR spectroscopy is used to detect the emergence of new interactions, marked by the appearance of new wave number shifts. As shown in Figure 4C, the strong absorption vibration peak at 3,414 cm^−1^ is attributed to the -OH stretching vibration of SA, and the smaller absorption peak at 2,936 cm^−1^ is attributed to the C-H stretching vibration absorption peak. 1,612 and 1,416 cm^−1^ are the asymmetric and symmetric stretching vibration of -COO-, which is the characteristic absorption peak of SA. 1,034 cm^−1^ is the C-O-C stretching vibration peak of pyran ring of SA. The broad absorption peak of LZM at 3,308 cm^−1^ is attributed to the stretching vibration peaks of the functional groups O-H and N-H. The characteristic absorption peaks of LZM at 1,660 cm^−1^ and 1,530 cm^−1^ are the amide I and amide II bands, respectively. The absorption peak at 1,642 cm^−1^ is caused by the -C=O stretching vibration or N-H bending vibration, while the absorption peak at 1,536 cm^−1^ is caused by the N-H stretching vibration. The main changes observed in the PC-SLC complex spectra compared to SA, LZM and PC are the O-H and amide bond stretching vibrational peaks located in the 1,500-1,660 cm^−1^ region. It is noteworthy that the formation of PC-SLC complexes was accompanied by a shift in the peak wave number of the hydrogen bonds, along with a shift change in the amide I and amide II bonds. The precise vibration of the carboxyl group (1,612 cm^−1^) in SA can no longer be observed, which may be attributed to the electrostatic interaction between the amino and carboxylate groups, leading to the merger of the two vibrations. These results all suggest that electrostatic interactions are the main force that enables the formation of the complex PC-SLC. Chang et al. (26) studied the IR spectra of Zein/NaCas/pectin complexes and found that the O-H vibrations and the amide bond region undergo major IR band changes in the formed complexes, and the carboxyl group vibrations of pectin disappear, all of which indicate that the formation of the complexes is driven by electrostatic driving force of the interaction.

#### SEM Analysis

The apparent morphology of PC-SLC complexes was observed using scanning electron microscopy and the results are shown in Figure 4D. The PC-SLC complex shows an irregular spherical structure and the spheres are clustered together. Yue et al. (27) showed that the ideal structure to form a complex is one with a uniform, smooth spherical surface with fewer cracks and collapses in the walls. It has also been shown that complex with rough surfaces are more sensitive to oxidation reactions due to the large surface area they possess, while complex with smooth surfaces are less susceptible to oxidation reactions (28). Therefore, it is assumed that the aggregated spheres may also increase the stability due to the reduction of surface area. The reason for the aggregation may be that the pressure during the freeze-drying process causes the spheres to squeeze each other together. Kurniasih et al. (29) microencapsulated chlorophyll with maltodextrin-fish gelatin and showed the formation of ice crystals during freeze-drying while generating porous forms due to sublimation processes.

### Stability of PC-SLC

#### pH Stability Analysis

**Figure 5:**
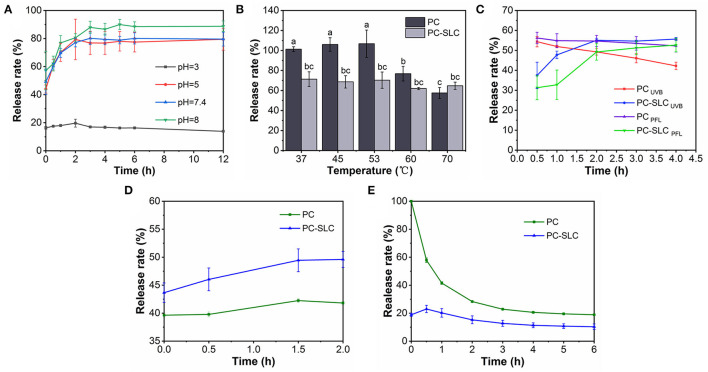
Stability of PC-SLC under pH **(A)**, temperature **(B)**, light **(C)**, simulated gastric fluid, **(D)** and simulated intestinal fluid **(E)** conditions.

Figure 5A shows the cumulative release rate of PC from complex PC-SLC in disodium hydrogen phosphate-citrate buffer solution at pH 3-8. The pH stability was evaluated by monitoring the change of PC release rate with time (0-12 h). The release of PC from the complexes under all pH conditions showed a two-step release profile, including a burst release within 2 h and a continuous slow release for 10 h. The reason for this may be due to the physical adsorption to the PC of PC-SLC and almost 90% of the PC has been released within 12 h. Zhang et al. (30) prepared polysaccharide supramolecular hydrogels based on carboxymethyl β-cyclodextrin/chitosan inclusion complexes and EDTA-chitosan constructs by electrostatic interactions. The system loading of BSA resulted in a continuous slow release of BSA in a buffer solution at pH 7.4 for 12 h, after which stability was achieved. The sustained release behavior may be attributed to the fact that PC-SLC form a barrier to protect the PC (31). Similar results were obtained from Khan et al. (8) by studying the controlled release in CA/NaAlg polyelectrolyte complexes (PEC). PC was essentially not released at pH = 3, while the highest release rate was observed at pH = 8. This indicates that the closer the pH value of the system is to alkalinity, the more unstable the complex is and the faster release of PC. This is because the isoelectric points of PC and LZM are 3.4 and 11, respectively, and both are positively charged at pH = 3 so that they cannot be bound by electrostatic force. PC and LZM, one negatively charged and one positively charged, form complex with SA in aqueous solution when the pH increases. PC is unstable under strong alkaline conditions, which may be responsible for the increased release rate. The efficacy of complexes generated by protein-polysaccharide interactions for nutrient encapsulation and colloidal stability was preliminarily demonstrated by Hosseini et al. by electrostatic interaction forces under changing pH conditions (32).

#### Thermal Stability Analysis

Xu et al. (33) studied the stability of PC at 30-70°C, and the general denaturation temperature of PC is 45°C. Therefore, this experiment investigated the release of PC and PC-SLC complexes in disodium hydrogen phosphate-citrate buffer solution at different temperature conditions, and the results are shown in Figure 5B. The results showed that the stability of PC decreased significantly above 53°C, while the prepared PC-SLC complexes showed higher stability than PC. This may be due to the protective effect on the stability of PC after the formation of complex. As Alehosseini et al. (34) studied the stabilization of Pickering emulsions by CSNPs to improve the thermal stability of D-limonene. Suzery et al. (35) prepared microencapsulated PC using sodium alginate as coating material by extrusion method. Microspheres with high encapsulation efficiency were prepared by varying the amount of sodium alginate. The stability of the microspheres was observed at hot air drying (43°C) and freezing (−2°C) conditions. It was found that the stability of the prepared microspheres was greatly influenced by different temperature conditions.

#### Photostability Analysis

PC is unstable under light conditions (36). In order to determine whether the complexes can increase the stability of PC under light conditions, this study investigated the stability of PC and PC-SLC in disodium hydrogen phosphate-citrate buffer solution at pH = 7.4 using UVB light. As shown in Figure 5C, the release of PC-SLC was faster under light conditions than under non-light conditions, and there was a tendency to lose PC. The end point of release was reached when the release lasted for 4 h. The cumulative release rate of the complexes with light at this endpoint was higher than that of the samples without light. This suggests that light accelerates the release of PC-SLC and has an effect on the stability of PC-SLC. The effect of epigallocatechin gallate (EGCG) on the physicochemical properties of PC was investigated by Yang et al. (37). Under 8 days of light storage conditions, the structure of PC changed from α-helical structure to β-sheeted structure. Combining PC with EGCG to form a complex improved the stability of PC. It can be seen that SA-LZM can encapsulate PC so that PC will not be degraded under UVB light conditions, thus protecting and improving the stability of PC.

#### *In vitro* Simulated Digestion Stability Analysis of PC-SLC

The stability of the complexes under simulated gastrointestinal fluid conditions was assessed by measuring the release rate of PC (Figures 5D,E). The samples PC and PC-SLC showed good stability with 42.24 ± 0.37 and 49.45 ± 2.03% release rates of PC in SGF, respectively. The relative release rate of PC-SLC remained stable within 2 h in SGF. When PC-SLC was incubated in SIF, the release rate increased rapidly to 23% within 30 min and remained relatively stable thereafter. This may be due to the fact that the formed PC-SLC protects the PC from pepsin digestion in the simulated gastric juice to some extent and is digested by trypsin in the simulated intestinal juice (38). Barbosa et al. (39) used the formation of composite gels of carboxymethylcellulose and whey protein isolated complex to encapsulate sachainchioil containing β-carotene (β-C), and the prepared microcapsules could be preserved under SGF conditions and showed higher release during *in vitro* simulated intestinal digestion. The complex is stabilized in the gastric juice and slowly digested in the intestinal juice, which is usually the most ideal situation (40). The above study showed that PC-SLC have the ability to deliver PC to the intestine.

### Analysis of Antioxidant Activity of PC-SLC

**Figure 6:**
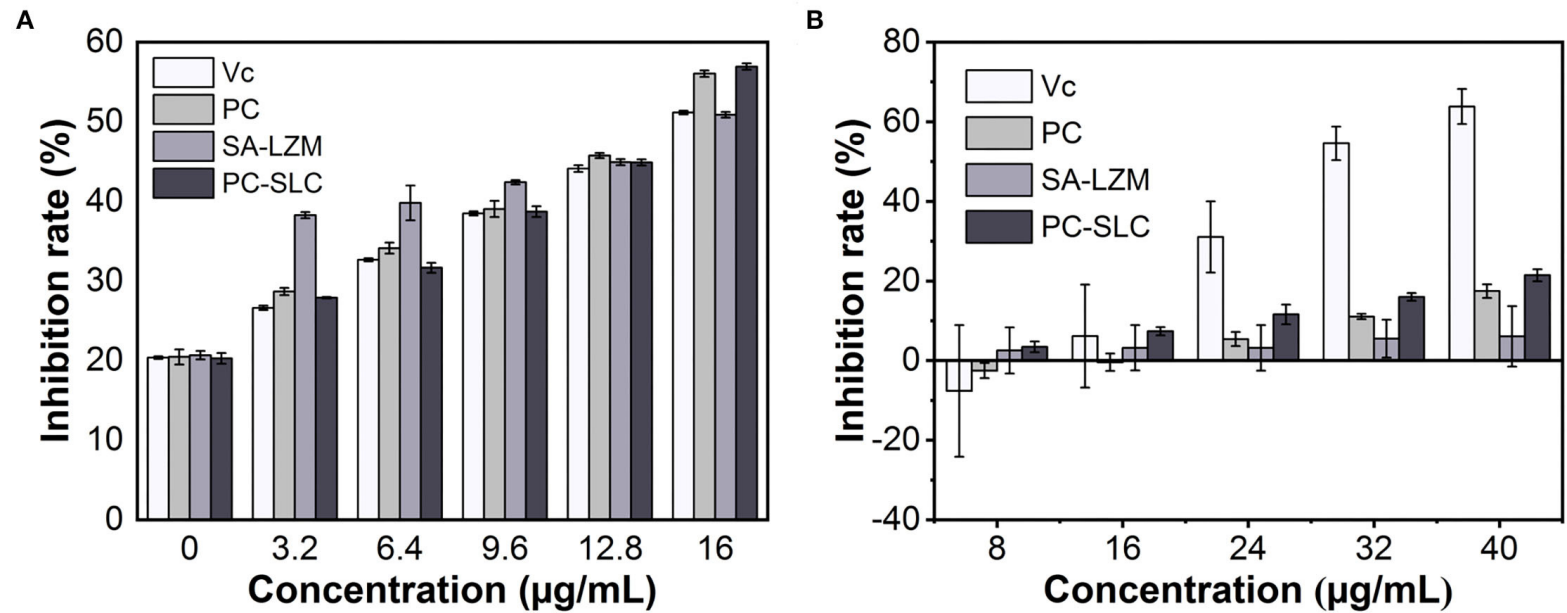
Determination of DPPH **(A)** and ABTS **(B)** radical scavenging capacity of the complex PC-SLC.

The antioxidant activity of PC is related to its unique structure (41). In this study, the antioxidant activity of PC-SLC was evaluated by ABTS and DPPH free radical scavenging. Figures 6A,B show the antioxidant capacity results of different samples at various concentrations. The results indicated that all samples showed significant antioxidant capacity. Schmatz et al. (42) prepared a novel nanofiber material, PC/polyvinyl alcohol nanoparticles (PC-PVAn), using electrostatic spinning and electrospraying techniques. Polymer nanofibers containing PC-PVAn showed antioxidant capacity in all methods used (ABTS, DPPH and reducing power). However, the antioxidant activity of the free PC-PVAn in the medium was significantly higher than that of the PC-PVAn incorporated into the nanofibers. This result was expected because using the same sample mass, the phycocyanin concentration present was at higher proportions in the nanoparticles compared to the nanofibers, and this may have been reflected in the quantification of the antioxidant capacity of phycocyanin. Similarly, the antioxidant capacity of PC-SLC obtained by adding the same concentration of sample solution in this study was slightly lower than that of free PC. In conclusion, the ABTS radical scavenging ability of PC-SLC was slightly better or not much different at the same sample concentration, but at the same time we observed that SA-LZM complex without PC also had certain antioxidant activity (43, 44). Therefore, it can be inferred that due to the addition of SA and LAM, the antioxidant activity of PC-SLC may be higher than that of PC.

As shown in Figure 6B, the DPPH radical scavenging ability of each sample increased gradually with the increase of the sample concentration. Generally, the DPPH radical scavenging ability of PC-SLC was higher than that of PC. The results showed that the formed complex PC-SLC had good DPPH radical scavenging ability.

The above antioxidant experimental results show that PC-SLC is a powerful natural antioxidant that can be better applied in various fields.

## Conclusion

In this study, PC-SLC complex were successfully prepared by electrostatic interactions between SA and LZM. The complexes formed at a mass ratio of 0.1 for SA-LZM showed the highest PC encapsulation efficiency (89.9 ± 0.374%). Then the stability and antioxidant activity of the complexes were explored. It was found that the formed complex PC-SLC has a protective effect on PC and can improve the stability of PC by studying the stability of the complex at different pH, temperature, light and simulated gastrointestinal fluid conditions. The good antioxidant activity of the complex PC-SLC was demonstrated by the ABTS and DPPH radical scavenging ability tests. These findings may expand the application of polysaccharide-protein complexes as new carriers of bioactive substances in drug delivery systems, as well as broaden the range of PC applications.

## Data Availability Statement

The original contributions presented in the study are included in the article/supplementary material, further inquiries can be directed to the corresponding author/s.

## Author Contributions

B-WQ: conceptualization, investigation, writing—original draft, and writing—review and editing. X-TL: investigation and writing—original draft. C-XW: data curation and investigation. SS and C-QA: conceptualization, methodology, and supervision. Y-HF: conceptualization, investigation, project administration, resources, software, and writing—review and editing. All authors contributed to the article and approved the submitted version.

## Funding

This research was supported by the National Natural Science Foundation of China (Grant No. 31501431) and the National Key Research and Development Program of China (2018YFD0901106).

## Conflict of Interest

The authors declare that the research was conducted in the absence of any commercial or financial relationships that could be construed as a potential conflict of interest.

## Publisher's Note

All claims expressed in this article are solely those of the authors and do not necessarily represent those of their affiliated organizations, or those of the publisher, the editors and the reviewers. Any product that may be evaluated in this article, or claim that may be made by its manufacturer, is not guaranteed or endorsed by the publisher.
